# Ependymal and neural stem cells are close relatives

**DOI:** 10.1016/j.stemcr.2025.102574

**Published:** 2025-07-03

**Authors:** Georgia Lokka, Anna Chantzara, Zoi Lygerou, Stavros Taraviras

**Affiliations:** 1 Department of Physiology, School of Medicine, University of Patras, Patras, Greece; 2 Department of General Biology, School of Medicine, University of Patras, Patras, Greece

**Keywords:** multiciliated ependymal cells, biciliated ependymal cells, neural stem cells, plasticity

## Abstract

Multiciliated ependymal and neural stem cells are key cell populations of the subventricular zone. Recent findings revealed that at least a subpopulation of radial glial cells during embryogenesis can be bipotent and produce both neural stem cells and ependymal cells. The balance between these cell populations is orchestrated by Geminin superfamily, ensuring optimal niche function. However, whether cell fate decisions are definitive or dynamic and whether potential regional differences exist remain elusive. In this review, we delve into the shared origins of different subventricular zone cell populations, and we explore the potential interplay among them. Moreover, we compile evidence on the de-differentiation capacity of ependymal cells and their controversial neural stem cell function under specific conditions, with emphasis on the possible implication of a rare population of biciliated (E2) ependymal cells. Understanding the mechanisms regulating cell fate decisions may unravel ependymal cells’ therapeutic potential in therapies targeting various human diseases.

## Teaser

Lokka et al. review the shared origins of subventricular zone cell populations, focusing on neural stem cells and ependymal cells, with Geminin family members regulating the balance between them. The review also addresses the controversial neural stem cell potential of ependymal cells, placing particular emphasis on an understudied population of biciliated (E2) ependymal cells.

## Introduction

Neural stem cells (NSCs) in the adult subventricular zone (SVZ) are primarily quiescent, and upon activation, they give rise to transit-amplifying progenitor cells, which subsequently generate neuroblasts. Neuroblasts migrate toward the olfactory bulbs and form neurons (Lim and Alvarez-Buylla 2014; 2016; Chaker et al., 2016; Obernier and Alvarez-Buylla 2019). Neurogenesis is tightly regulated to ensure the preservation of an adequate number of stem cells throughout life (Lim and Alvarez-Buylla 2016; Obernier and Alvarez-Buylla 2019; Urbán and Cheung 2021).

While the aforementioned cell populations are directly implicated in neurogenesis, the SVZ also contains ependymal cells (ECs) that interact closely with NSCs and indirectly impact neuron formation. ECs contribute to cerebrospinal fluid (CSF) circulation and molecule secretion, affecting the signals NSCs receive from their environment (Lim and Alvarez-Buylla 2014; Spassky and Meunier, 2017; Obernier and Alvarez-Buylla 2019).

However, the molecular pathways underlying the differentiation of distinct EC subtypes remain largely unexplored. Notably, the stem cell potential and neurogenic capacity of ECs remain subjects of ongoing debate. Conflicting data in the literature suggest that ECs may have a more intricate role in neurogenesis than previously thought. In this review, we synthesize the current knowledge to address these questions and identify the areas worth further investigation.

## Specification of ECs during embryogenesis

Previous studies have demonstrated that ECs derive from a subpopulation of radial glial cells committed toward this lineage during mid-embryogenesis (Merkle et al., 2004, Spassky et al., 2005; Ortiz-Álvarez et al., 2019). More specifically, in early embryogenesis, neuroepithelial cells of the neural tube undergo symmetric divisions expanding the progenitor pool, and at embryonic day E9.5–E10.5, they transition into radial glial cells (Misson et al., 1988). Then, radial glial cells commit to the EC fate during embryonic days E14.5–E16.5 (Spassky et al., 2005). While commitment is initiated during embryogenesis, differentiation toward ECs occurs postnatally. As cells lose radial glial characteristics, they start generating basal bodies and forming densely packed cilia (Spassky et al., 2005). Notably, at birth, motile cilia can only be detected in the medial wall of the lateral ventricles, while in the lateral walls multiciliated cells will subsequently differentiate (Spassky et al., 2005; Abdelhamed et al., 2018). Differentiation of multiciliated ECs occurs along the posterior-to-anterior and ventral-to-dorsal axes of the lateral ventricular walls (Spassky et al., 2005; Kyrousi et al., 2017).

The specification of ECs occurs in a tightly regulated spatiotemporal manner. BrdU labeling experiments and S100b expression in mouse embryos identified double-positive cells in the caudal regions as early as E12, with their presence progressively extending rostrally by E14 and E18, indicating a caudal-to-rostral expansion of ECs (Spassky et al., 2005). Moreover, the expression of p73 and Foxj1, two key transcription factors, is essential for EC differentiation. Initially, Foxj1 expression is restricted in posterior regions and is gradually expanded anteriorly (Kyrousi et al., 2015). Foxj1 expression is maintained through embryogenesis, with the majority of cells lining the wall of the lateral ventricles expressing Foxj1 at E18.5. However, Foxj1 and p73 expression is gradually reduced postnatally (Pozniak et al., 2002; Kyrousi et al., 2015; Gonzalez-Cano et al., 2016; Fujitani et al., 2017; Abdi et al., 2018). The expression pattern of S100β also proposes a posterior-to-anterior expansion of ECs in the first two postnatal weeks. By the end of this period, the maturation of ECs is completed, and they are considered post-mitotic (Spassky et al., 2005; Kyrousi et al., 2017). Their apical surface is expanded up to 11-fold (Redmond et al., 2019) and forms rosette-like structures with NSCs in the walls of the lateral ventricles.

During development, ECs adopt regionally distinct characteristics, driven by dynamic gene expression and region-specific proliferation. In the lateral ventricles, ECs are predominantly multiciliated and play a key role in supporting the NSC niche. In the third and fourth ventricles of the brain, along with multiciliated ECs, an enriched population of biciliated (E2) ECs is observed (Mirzadeh et al., 2017). In these regions, ECs aid CSF flow and interact with the choroid plexus to support barrier and secretory functions (Ji et al., 2022). Within the spinal cord, ECs that line the central canal typically exhibit a biciliated morphology, while it has been proposed that they retain latent stem cell potential (Stenudd et al., 2022). Despite their spatially distinct functions, ECs collectively support CSF dynamics and contribute to the structural and functional maturation of the central nervous system. However, the variability in ciliary number among EC subpopulations and their region-specific distribution throughout the ventricular system suggest a potential functional specialization.

## Exploring the characteristics of SVZ ependymal cell subtypes

**Figure 1:**
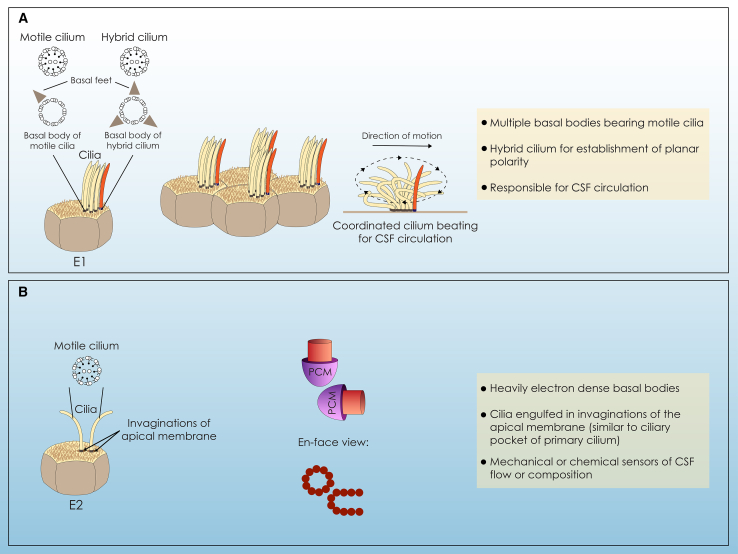
Subtypes of ECs in the SVZ (A) Multiciliated ECs (E1) bear multiple motile cilia on their apical surface. Each motile cilium consists of 9 pairs of microtubules surrounding a central pair (9 + 2 structure) and is anchored by a basal body with a single basal foot. A distinctive cilium has also been identified, with a 9 + 2 structure and a basal body that possesses multiple basal feet, resembling the basal body of a primary cilium, therefore called a hybrid cilium. The hybrid cilium is necessary for the coordinated beating of motile cilia, supporting the appropriate CSF flow. (B) Biciliated (E2) ECs possess one to two motile cilia, arising from orthogonally oriented basal bodies that are engulfed in invaginations of their apical membrane, resembling the ciliary pocket of the primary cilium. It has been proposed that they act as mechanical or chemical sensors of the CSF.

In the SVZ, multiciliated ECs (E1) bear on their apical surface approximately 50 motile cilia (Mirzadeh et al., 2008) (Figure 1A). Each motile cilium has nine pairs of microtubules that surround a central pair (9+2 structure) and is anchored to the apical EC surface by a modified centriole called a basal body (Ishikawa 2017; Soares et al., 2019). The large number of basal bodies arises through the activation of two main pathways during multiciliated EC differentiation: the parental centriole-dependent pathway and the deuterosome-dependent pathway (Zhao et al., 2019; Mahjoub et al., 2022). The deuterosome-dependent pathway, also called the “*de novo*” pathway, differs from the parental centriole-dependent pathway as it does not rely on pre-existing centrioles. Instead, it organizes multiple new centrioles around non-microtubule structures known as deuterosomes and is responsible for 95% of the centriole population in ciliated cells (Shahid and Singh, 2018). Each basal body in multiciliated ECs possesses a single basal foot (Figure 1A), essential for the proper orientation and organization of cilia (Soares et al., 2019). More specifically, upon basal body amplification, basal bodies align in rows, having their basal feet oriented toward the direction that cilia move, thus defining the orientation of planar ciliary beating (Figure 1A) (Spassky and Meunier 2017; Ryu et al., 2021).

Recently, a specialized cilium with characteristics of both primary and motile cilia, known as a hybrid cilium, was identified as fundamental for basal body alignment in multiciliated ependymal and airway cells (Liu et al., 2020). The hybrid cilium has a central pair of microtubules and proteins associated with the ciliary beating machinery of motile cilia, while it is anchored by a basal body with multiple basal feet, resembling that of a primary cilium (Figure 1A). Interestingly, pulse-chase experiments, commonly used for the discrimination between centrosomal centrioles and newly synthesized basal bodies of ECs, demonstrated that the basal body anchoring the hybrid cilium derives from the parental centrioles (Liu et al., 2020). Although the hybrid cilium is necessary for the appropriate organization of the rest of motile cilia, thus affecting the overall functionality of ECs, the mechanism underlying its formation remains elusive. It has been proposed that it is different at least in part from the mechanism governing the formation of remainder motile cilia, as it can be detected in patients with CCNO mutations that lack multiciliated cells (Liu et al., 2020).

Cilia beat coordinately contributing to CSF flow, which is fundamental for homeostasis maintenance and subsequently for proper mouse brain function (Del Bigio, 2010; Siyahhan et al., 2014; Meunier and Azimzadeh, 2016; Spassky and Meunier, 2017). Defects in EC generation or function have been related to hydrocephalus development in mice, characterized by the accumulation of CSF into the ventricular system (Banizs et al., 2005; Del Bigio 2010; Ohata et al., 2014; Kumar et al., 2021; Yamada et al., 2021). Mutations in genes associated with multiciliated EC generation can lead to hydrocephalus development in humans as well (Amirav et al., 2016; Robson et al., 2020; Hochstetler et al., 2022; Hou et al., 2023). This underscores the importance of unraveling the molecular pathway underlying multiciliated EC differentiation, a highly conserved mechanism in multiciliated cells of different systems and different organisms, including humans (Spassky and Meunier, 2017; Lewis and Stracker, 2021).

While multiciliated ECs have garnered attention due to their important role, the subpopulation of ECs with one or two long cilia (biciliated, E2) (Figure 1B) has not been widely explored. Biciliated ECs represent approximately 5% of cells in the SVZ, while they are more abundant in the third and fourth ventricles of the brain (Mirzadeh et al., 2017). They also have many common features with biciliated ECs in the central canal of the spinal cord (Eccs) (Alfaro-Cervello et al., 2012); however, it is not clear whether they correspond to the same cell population.

To delve deeper into their features, biciliated ECs have two basal bodies with a unique morphology, constituting their most notable structural hallmark. More specifically, they are larger than those of multiciliated ECs and form characteristic donut or horseshoe-like structures due to their vertical orientation (Figure 1B). When compared with multiciliated ECs, biciliated ECs have a smaller apical surface and deeper interdigitations of their membrane, with long lateral extensions. Furthermore, in multiciliated ECs, mitochondria are in close proximity to their basal bodies, while in biciliated ECs, mitochondria are located around the nucleus. Interestingly, in contrast with motile cilia of multiciliated ECs, cilia of biciliated ECs are engulfed in invaginations of their apical membrane (Figure 1B), a trait reminiscent to the ciliary pocket observed in the primary cilium (Mirzadeh et al., 2008).

The two cilia of biciliated ECs are unlikely to contribute to the CSF flow, so it has been proposed that their role differs from that of multiciliated ECs. The structure of basal bodies and the invaginations from which cilia protrude advocate that they may serve as mechanical or chemical sensors for monitoring the flow and composition of CSF. The intricate basal bodies of biciliated ECs can potentially contribute to signal transmission between the axoneme of cilia and intracellular compartments (Mirzadeh et al., 2017). Understanding the role of biciliated ECs in the brain is a subject of considerable interest. Their presence in higher numbers in regions critical for overall brain function suggests they may possess regulatory properties. More specifically, in the third ventricle, it has been proposed that biciliated ECs correspond to tanycytes, suggesting a potential role as neural progenitors for hypothalamic neurons involved in energy balance control and in modulating access of metabolic signals to this region (Mirzadeh et al., 2017). In the fourth ventricle, their location over the raphe nuclei suggests that they may cooperate with serotonergic neurons that control neurogenesis (Mirzadeh et al., 2017).

## The shared lineage of NSCs and ECs and the regulatory role of Geminin family members

**Figure 2:**
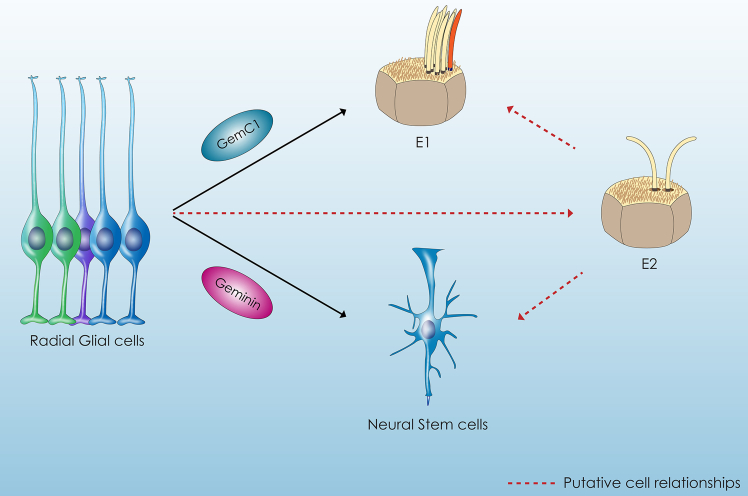
ECs and NSCs have a common origin Multiciliated ECs and adult NSCs derive from common neural progenitors, radial glial cells. GemC1 is necessary for ependymal cell generation while Geminin favors the formation of NSCs, indicating that Geminin family members coordinate the balance of these cell populations. However, the potential of biciliated (E2) ECs to act as NSCs or give rise to multiciliated ECs under specific conditions remains elusive.

In order to sustain neurogenesis throughout adult life, quiescence, self-renewal and differentiation are processes of high importance that need to be strictly regulated through symmetric or asymmetric divisions (Obernier and Alvarez-Buylla, 2019). The commitment of adult NSCs begins in early embryogenesis, at E9.5, from a p57-expressing subpopulation of slowly dividing neural progenitor cells (Furutachi et al., 2015). Moreover, it has been reported that some NSC precursors are generated between E13.5 and E15.5, with their specification evident as early as E11.5 (Fuentealba et al., 2015). Although the temporal window of adult NSCs coincides with that of ECs, a link between these two populations has only recently been revealed. Redmond et al., 2019 used lineage tracing methods, which highlighted the presence of clonally related ECs and adult NSCs, indicating that at least a subpopulation of radial glial cells has the potential to generate both cell types (Redmond et al., 2019). In addition, high-resolution lineage tracing experiments by Ortiz-Álvarez et al. (2019) supported the hypothesis that ECs and adult NSCs derive from a common progenitor radial glial cell committed at E13.5–E15.5. They also proposed that these cell populations are generated sequentially: most adult NSCs are produced earlier through symmetric or asymmetric divisions, whereas the majority of ECs are formed later through terminal symmetric divisions (Ortiz-Álvarez et al., 2019). Furthermore, they revealed the important regulatory role of Geminin family members during this process. More specifically, clonal analysis experiments showed that GemC1 overexpression leads to cell cycle exit, formation of smaller clones, and premature formation of ECs through symmetric divisions. On the other hand, Geminin favors the formation of clones that through symmetric divisions produce adult NSCs (Figure 2) (Ortiz-Álvarez et al., 2019).

Geminin family is composed of Geminin, GemC1, and McIdas, three evolutionarily related molecules bearing a conserved coiled-coil domain, that enables the formation of homodimers or heterodimers (Pefani et al., 2011). The interactions of Geminin family members are fundamental for orchestrating the balance between DNA replication initiation and differentiation in various tissues (Patmanidi et al., 2017; Arbi et al., 2018). Geminin has been proposed to play a crucial role in cell fate decisions (Karamitros et al., 2011; Spella et al., 2011; Patmanidi et al., 2017) in nervous and hematopoietic system, by regulating transcription and chromatin organization (Karamitros et al., 2010; 2015; Spella et al., 2011; Konstantinidou et al., 2016; Patmanidi et al., 2017). GemC1 with homology to Geminin in the coil-coil domain was identified for its participation in the formation of the pre-initiation complex (Balestrini et al., 2010), while McIdas was first described as a protein that binds and inhibits Geminin function (Pefani et al., 2011; Caillat et al., 2013). Both genes are transiently expressed from mid- to late embryogenesis in periventricular zone cells, in a posterior-to-anterior and ventral-to-dorsal pattern. GemC1 is the most upstream known regulator of multiciliogenesis mainly based on its early expression pattern and its ability to activate McIdas expression (Kyrousi et al., 2017; Spassky and Meunier 2017; Arbi et al., 2018). GemC1 and McIdas activate the transcription of key transcription factors for ependymogenesis, including p73, Foxj1, and c-myb. Through this regulation, they control the expression of the full repertoire of genes characterizing mature ECs, including those required for basal body amplification and docking, as well as ciliary anchoring (Kyrousi et al., 2015; Arbi et al., 2016; Spassky and Meunier 2017). Notably, McIdas can rescue the ependymogenesis program when ectopically expressed in radial glial cells lacking GemC1 expression suggesting a functional redundancy between the two genes (Kaplani et al., 2024).

Interestingly, GemC1 knockout mice revealed that cells in the SVZ, instead of differentiating toward ECs, acquire characteristics of adult NSCs (Lalioti et al., 2019b). More specifically, Lalioti et al. showed that in GemC1 knockout mice, an increased expression of GFAP and BLBP is detected. Furthermore, single-cell deletion of GemC1 in mice show that upon GemC1 deletion, increased number of cells located next to the ventricular lumen express GFAP and are in contact with blood vessels and show Ashl1 and Pax6 expression, suggesting that these cells adopt a neurogenesis pathway. These *in vivo* data further reinforce the fate-mapping data of a common progenitor for ECs and adult NSCs. Furthermore, assay for transposase-accessible chromatin sequencing experiments showed that cells isolated from GemC1-deficient mice adopt a more “open” chromatin organization in genes identified in NSCs, proposing a global reorganization linked to adult NSC phenotype emphasizing the key role of GemC1 as a pioneering factor for the initiation of the ependymal fate (Lalioti et al., 2019b). Mechanistically, ependymogenesis is likely to be regulated by the ability of GemC1 and McIdas to regulate transcription and chromatin organization, through complexes that are formed by E2F4/5, DP1, and BAF (Ma et al., 2014; Arbi et al., 2016; Terré et al., 2016; Lalioti et al., 2019a; Lewis et al., 2023), while lack of GemC1 pushes cells to choose the adult NSC pathway as the default.

However, the role of Geminin family members in the generation of distinct cell populations within the SVZ remains partially elucidated as these molecules function preferably as heterodimers, while their function might not be limited only to transcriptional regulation.

## Definitive determination or dynamic cell state?

The shared origin of NSCs and ECs in the SVZ raises questions about the possible NSC potential of ECs. Initial experimental approaches, involving administration of BrdU or [3H] thymidine in adult mice, demonstrated that ECs lining the brain ventricles have non-dividing nature under normal conditions (Doetsch et al. 1999a, 1999b; Spassky et al., 2005), with subsequent studies substantiating these findings (Carlén et al., 2009; Shah et al., 2018). Furthermore, several publications argue that ECs lack key characteristics of NSCs, such as the ability to self-renew and sustain their own population (Spassky et al., 2005; Carlén et al., 2009; Shah et al., 2018). However, some publications supported that ECs can proliferate and produce neurons *in vivo* (Johansson et al., 1999; Coskun et al., 2008; Nomura et al., 2010; Luo et al., 2015; Stratton et al., 2019) or that they can generate neurons and astrocytes, only under specific conditions, such as upon brain injury or exposure to growth factors (Johansson et al., 1999; Li et al., 2002; Carlén et al., 2009; Nomura et al., 2010; Luo et al., 2015; Shah et al., 2018).

Conflicting data regarding the NSC potential of ECs arise due to two main factors: (1) non-specific labeling strategies were used in lineage tracing experiments, and (2) there were difficulties in isolating distinct EC populations, thus impacting reliability. Consequently, it was very important to develop innovative EC tracing methods, designed to specifically label ECs and overcome previous limitations that obstructed the acquisition of conclusive results. In this context, fluorescence-activated cell sorting (FACS)-purified CD24^+^ ECs were isolated and then were cultured under neurosphere-generating conditions. ECs were unable to proliferate and form neurospheres, and they lacked the expression of the unique NSC marker, Lewis X (Capela and Temple, 2002). Toward the same direction, a novel transgenic fluorescent system driven by alpha smooth muscle actin was utilized for EC labeling, offering enhanced specificity over previously adopted methods. Labeled ECs isolated through FACS were unable to proliferate both under classic neurosphere-forming conditions and after vascular endothelial growth factor (VEGF)/fibroblast growth factor addition, while they were tested negative for VEGFR1. From the opposing perspective, single-cell sequencing experiments demonstrated that the cluster of cells expressing VEGFR1 (Flt1) in this study lacked the expression of EC cilia-related genes (Shah et al., 2018). These findings reinforce the non-proliferative nature of ECs, challenging previous single-cell transcriptome data supporting that ECs express VEGFR and can be activated by VEGF (Luo et al., 2015).

Furthermore, single-cell transcriptomic analysis on FACS-purified cells showed that ECs have a unique transcriptional profile, enriched in genes related to cilia formation. Even though they share 2,375 genes with NSCs due to their common origin, only 193 genes were unique to ECs, while 128 were specific for NSCs. The genes that ECs share with quiescent NSCs and activated NSCs are related to neural development and microtubule-associated processes, respectively. However, the signature of mature ECs was unique and characterized by numerous gene sets associated with cilia, creating a distinct profile that clearly differentiated them from the entire lineage of neural stem and progenitor cells while no functional stemness could be detected in ECs (Shah et al., 2018).

A remarkable finding was that ECs can de-differentiate and enter the cell cycle under certain circumstances. More specifically, it was shown that in order to remain differentiated, ECs need to retain the levels of Foxj1, while Foxj1 degradation results in de-differentiation and development of hydrocephalus (Abdi et al., 2018). In greater detail, Foxj1^CreER^^T^^2/+^; R26R-tdTomato mice were used to detect ECs and their progeny, showing that ECs can re-enter cell cycle and give rise to more ECs only under proliferating conditions (Abdi et al., 2018). Moreover, Foxj1^C^^reERT2::GFP^ mice, utilized for lineage tracing of ECs and their progeny, demonstrated that, in response to acute injury or stroke, they do not function as stem cells contributing to neurogenesis or gliogenesis (Muthusamy et al., 2018).

Overall, there is an ongoing debate regarding the stem cell properties of ECs with conflicting data, making it hard to reach substantiated conclusions. Variations in mouse strains, age, and methods may affect the detectability of mitotic characteristics in the ependyma, either enhancing or diminishing their visibility. However, recent publications utilizing more sensitive methods and single-cell transcriptomic analysis support that ECs need constant signaling to remain differentiated. Although the current knowledge does not provide strong evidence for the acquisition of NSC characteristics, it cannot be completely excluded that, at a specific stage during EC fate determination, the acquisition of NSC properties may occur.

## Biciliated ECs: Potential activation and implication in brain damage repair

The controversial plasticity of ECs in the brain is further corroborated by some characteristics of ECs in the spinal cord. More specifically, the central canal of the spinal cord is lined with biciliated ECs (Eccs), which carry two invaginated long motile cilia (9+2), and their basal bodies have electron-dense pericentriolar material—key features they share with biciliated (E2) ECs in the brain. In addition, the cells in the central canal expressing Foxj1 also express CD24 and have two donut-shaped basal bodies, similar to biciliated (E2) ECs in the brain (Alfaro-Cervello et al., 2012).

It is commonly accepted that spinal cord ECs are rarely proliferating under normal conditions. However, [3H]thymidine and BrdU labeling revealed that biciliated ECs were the most commonly proliferating cell type during spinal cord growth (Alfaro-Cervello et al., 2012). Furthermore, many studies have shown that some ECs in the spinal cord can be activated after injury and can take part in the regenerative processes in animal models, with a few oligodendrocytes and mainly astrocytes being formed *in vivo* (Furube et al., 2020; Chevreau et al., 2021; Stenudd et al., 2022; Rodriguez-Jimenez et al., 2023). Concurrently, induction of spinal cord ECs *in vitro* leads to neurosphere formation and neuron generation, indicating that they acquire neurogenic potential (Meletis et al., 2008; Barnabé-Heider et al., 2010).

The high heterogeneity of cells within the central canal of the spinal cord has contributed to acquiring conflicted data in the past regarding their identity and function. However, the recent development of innovative methodologies for characterizing distinct cell populations has paved the way for a more comprehensive understanding of the dynamics in these cellular populations (Milich et al., 2021). For example, a subpopulation of ECs in the spinal cord, characterized by the expression of Troy (also called EpA), undergoes significant transcriptional changes after injury. More specifically, EpA cells lose the expression of ependymal-related genes and acquire a stem cell-like transcriptional profile, enabling their differentiation toward oligodendrocytes and astrocytes, as demonstrated by single-cell transcriptome analysis (Stenudd et al., 2022). Similarly, single-cell transcriptomic analysis of spinal cord ECs in adult mice identified some cells that can re-enter cell cycle and become immature post injury, supporting their possible role as spinal cord NSCs (Rodrigo Albors et al., 2023).

Despite these findings, the extent of EC contribution to progeny upon spinal cord injury remains a topic of debate (Ren et al., 2017). Provided that spinal cord ECs in the spinal cord resemble biciliated (E2) ECs of the brain, it is worth wondering whether these cell populations are indeed the same. The similarity between them also prompts the question of whether biciliated (E2) ECs in the brain could constitute a population capable of being activated and giving rise to other cell types during adult life, in a similar way to how spinal cord ECs may be activated following spinal cord injury.

## Areas requiring further investigation

Despite recent advances in understanding NSC and EC lineage and differentiation, several unresolved questions remain regarding the timing, regional specificity, and lineage relationships among these populations. A key area of uncertainty lies in the developmental trajectories of radial glial cells across distinct brain regions. Progenitor cells in the lateral ganglionic eminence (LGE) appear to follow “a set-aside model,” where adult NSCs are specified during embryogenesis but remain quiescent until postnatal differentiation (Furutachi et al., 2015). In contrast, their counterparts in the pallium exhibit a more extended commitment window, lasting into early postnatal stages, consistent with a “continuous” model of adult NSC generation (Marcy et al., 2023; Foucault et al., 2024). These findings underscore region-specific modes of adult NSC generation during development. Due to the shared origin of NSCs and ECs, it is plausible that pallial and LGE-derived ECs may also exhibit distinct temporal dynamics. It remains an open question whether a subset of ECs can be generated continuously, similar to pallial NSCs. Further investigation is required to determine whether this pattern is conserved across neurogenic regions or reflects region-specific variability that remains poorly understood.

Another intriguing question is whether biciliated ECs of the SVZ also share a common ancestry with multiciliated ECs and NSCs. Lineage tracing has shown that biciliated ECs in the third and fourth ventricles of the brain derive from the floor plate (Mirzadeh et al., 2017). However, their origin in the SVZ remains unclear. Understanding the mechanisms regulating the formation of biciliated ECs, especially how the balance of the different SVZ populations is orchestrated, is a captivating area of research.

A possible molecular mechanism controlling biciliated EC formation may involve the regulation of Foxj1 levels. Foxj1 is fundamental during multiciliogenesis for basal bodies’ docking on the apical surface of differentiating cells (Gomperts et al., 2004; Yu et al., 2008). Moreover, in *Xenopus* and zebrafish, it has been shown that Foxj1 has a pivotal role in the formation of motile node-like cilia and the generation of biciliated cells. Varying Foxj1 levels result in the formation of different subtypes of cilia: low levels lead to node-like cilia, higher levels result in two cilia, while the presence of additional regulators is required for multiciliogenesis (Stubbs et al., 2008). These results indicate that Foxj1 levels could also be crucial for biciliated cell formation in the mammalian brain and that cooperation with other factors may govern the balance between multiciliated and biciliated ECs.

Although biciliated ECs constitute a very small population, their presence in higher numbers in the third and fourth ventricles, which are important regulatory areas of the brain, suggests that these cells may be essential for maintaining brain homeostasis (Mirzadeh et al., 2017). Provided that biciliated ECs in the spinal cord are thought to be implicated in regeneration after injury, it would be interesting to investigate whether biciliated (E2) ECs constitute a subpopulation of ECs with similar properties in the brain. To specify further, it would be interesting to investigate whether the less-studied biciliated (E2) ECs have NSC potential or if they constitute intermediate neural progenitors capable of giving rise to adult NSCs and ECs in the brain (Figure 2).

Gaining insight into the function of biciliated ECs in the mouse brain would offer a potent tool for the development of innovative treatments. Recently, astrocytes were successfully reprogrammed toward multiciliated ECs, rendering them valuable for the development of hydrocephalus treatments for humans in the future (Kaplani et al., 2024). Similar approaches could be explored for the use of biciliated ECs of the SVZ and to transform them into fully differentiated multiciliated ECs or adult NSCs. This might constitute a very promising approach to treat hydrocephalus, brain injury, and neurodegenerative diseases.

## Acknowledgments

The authors thank Dr. Christina Kyrousi, Dr. Maria-Eleni Lalioti, and Dr. Konstantina Kaplani for their invaluable feedback on earlier drafts of this manuscript and their help in shaping the manuscript.

Financial support for this study was provided by the Hellenic Foundation for Research and Innovation (HFRI) under the “3rd Call for HFRI PhD Fellowships (Fellowship Νumber: 6674)”, awarded to G.L. The study was supported by Hellenic Foundation for Research and Innovation (H.F.R.I.) under the “2nd Call for H.F.R.I. Research Projects to support Faculty Members & Researchers” (Project Number: 2735), the European Union’s Horizon Europe (2021-2027), “ESPERANCE” ERA Chair program (GA 101087215) and HORIZON-WIDERA-2023-ACCESS-04 Pathways to Synergies “MILESTONE” project (GA 101159708; 10.3030/101159708) to S.T. The publication fees of this manuscript have been financed by the Research Council of the University of Patras.

## Author contributions

G.L. compiled literature research, wrote the manuscript, and produced the figures. A.C. reviewed and edited the manuscript. Z.L. reviewed and edited the manuscript. S.T. conceptualized, reviewed, and edited the manuscript. All authors read and approved the final manuscript as submitted.

## Declaration of interests

The authors declare no competing interests.

## References

[bib1] Abdelhamed Z., Vuong S.M., Hill L., Shula C., Timms A., Beier D., Campbell K., Mangano F.T., Stottmann R.W., Goto J. (2018). A mutation in Ccdc39 causes neonatal hydrocephalus with abnormal motile cilia development in mice. Dev. Camb. Engl..

[bib2] Abdi K., Lai C.-H., Paez-Gonzalez P., Lay M., Pyun J., Kuo C.T. (2018). Uncovering inherent cellular plasticity of multiciliated ependyma leading to ventricular wall transformation and hydrocephalus. Nat. Commun..

[bib3] Alfaro- Cervello C., Soriano-Navarro M., Mirzadeh Z., Alvarez-Buylla A., Garcia-Verdugo J.M. (2012). Biciliated ependymal cell proliferation contributes to spinal cord growth. J. Comp. Neurol..

[bib4] Amirav I., Wallmeier J., Loges N.T., Menchen T., Pennekamp P., Mussaffi H., Abitbul R., Avital A., Bentur L., Dougherty G.W. (2016). Systematic Analysis of CCNO Variants in a Defined Population: Implications for Clinical Phenotype and Differential Diagnosis. Hum. Mutat..

[bib5] Arbi M., Pefani D.-E., Kyrousi C., Lalioti M.-E., Kalogeropoulou A., Papanastasiou A.D., Taraviras S., Lygerou Z. (2016). GemC1 controls multiciliogenesis in the airway epithelium. EMBO Rep..

[bib6] Arbi M., Pefani D.-E., Taraviras S., Lygerou Z. (2018). Controlling centriole numbers: Geminin family members as master regulators of centriole amplification and multiciliogenesis. Chromosoma.

[bib7] Balestrini A., Cosentino C., Errico A., Garner E., Costanzo V. (2010). GEMC1 is a TopBP1-interacting protein required for chromosomal DNA replication. Nat. Cell Biol..

[bib8] Banizs B., Pike M.M., Millican C.L., Ferguson W.B., Komlosi P., Sheetz J., Bell P.D., Schwiebert E.M., Yoder B.K. (2005). Dysfunctional cilia lead to altered ependyma and choroid plexus function, and result in the formation of hydrocephalus. Dev. Camb. Engl..

[bib9] Barnabé-Heider F., Göritz C., Sabelström H., Takebayashi H., Pfrieger F.W., Meletis K., Frisén J. (2010). Origin of New Glial Cells in Intact and Injured Adult Spinal Cord. Cell Stem Cell.

[bib10] Caillat C., Pefani D.E., Gillespie P.J., Taraviras S., Blow J.J., Lygerou Z., Perrakis A. (2013). The Geminin and Idas coiled coils preferentially form a heterodimer that inhibits Geminin function in DNA replication licensing. J. Biol. Chem..

[bib11] Capela A., Temple S. (2002). LeX/ssea-1 Is Expressed by Adult Mouse CNS Stem Cells, Identifying Them as Nonependymal. Neuron.

[bib12] Carlén M., Meletis K., Göritz C., Darsalia V., Evergren E., Tanigaki K., Amendola M., Barnabé-Heider F., Yeung M.S.Y., Naldini L. (2009). Forebrain ECs are Notch-dependent and generate neuroblasts and astrocytes after stroke. Nat. Neurosci..

[bib13] Chaker Z., Codega P., Doetsch F. (2016). A mosaic world: puzzles revealed by adult neural stem cell heterogeneity. Wiley Interdiscip. Rev. Dev. Biol..

[bib14] Chevreau R., Ghazale H., Ripoll C., Chalfouh C., Delarue Q., Hemonnot-Girard A.L., Mamaeva D., Hirbec H., Rothhut B., Wahane S. (2021). RNA Profiling of Mouse ECs after Spinal Cord Injury Identifies the Oncostatin Pathway as a Potential Key Regulator of Spinal Cord Stem Cell Fate. Cells.

[bib15] Coskun V., Wu H., Blanchi B., Tsao S., Kim K., Zhao J., Biancotti J.C., Hutnick L., Krueger R.C., Fan G. (2008). CD133+ neural stem cells in the ependyma of mammalian postnatal forebrain. Proc. Natl. Acad. Sci. USA.

[bib16] Doetsch F., Caillé I., Lim D.A., García-Verdugo J.M., Alvarez-Buylla A. (1999). Subventricular zone astrocytes are neural stem cells in the adult mammalian brain. Cell.

[bib17] Doetsch F., García-Verdugo J.M., Alvarez-Buylla A. (1999). Regeneration of a germinal layer in the adult mammalian brain. Proc. Natl. Acad. Sci. USA.

[bib18] Del Bigio M.R. (2010). Ependymal cells: biology and pathology. Acta Neuropathol..

[bib19] Foucault L., Capeliez T., Angonin D., Lentini C., Bezin L., Heinrich C., Parras C., Donega V., Marcy G., Raineteau O. (2024). Neonatal brain injury unravels transcriptional and signaling changes underlying the reactivation of cortical progenitors. Cell Rep..

[bib20] Fuentealba L.C., Rompani S.B., Parraguez J.I., Obernier K., Romero R., Cepko C.L., Alvarez-Buylla A. (2015). Embryonic Origin of Postnatal Neural Stem Cells. Cell.

[bib21] Fujitani M., Sato R., Yamashita T. (2017). Loss of p73 in ependymal cells during the perinatal period leads to aqueductal stenosis. Sci. Rep..

[bib22] Furube E., Ishii H., Nambu Y., Kurganov E., Nagaoka S., Morita M., Miyata S. (2020). Neural stem cell phenotype of tanycyte-like ependymal cells in the circumventricular or-gans and central canal of adult mouse brain. Sci. Rep..

[bib23] Furutachi S., Miya H., Watanabe T., Kawai H., Yamasaki N., Harada Y., Imayoshi I., Nelson M., Nakayama K.I., Hirabayashi Y., Gotoh Y. (2015). Slowly dividing neural progenitors are an embryonic origin of adult NSCs. Nat. Neurosci..

[bib24] Gomperts B.N., Gong-Cooper X., Hackett B.P. (2004). Foxj1 regulates basal body anchoring to the cytoskeleton of ciliated pulmonary epithelial cells. J. Cell Sci..

[bib25] Gonzalez-Cano L., Fuertes-Alvarez S., Robledinos-Anton N., Bizy A., Villena-Cortes A., Fariñas I., Marques M.M., Marin M.C. (2016). p73 is required for ependymal cell maturation and neurogenic SVZ cytoarchitecture. Dev. Neurobiol..

[bib26] Hochstetler A., Raskin J., Blazer-Yost B.L. (2022). Hydrocephalus: historical analysis and considerations for treatment. Eur. J. Med. Res..

[bib27] Hou C.C., Li D., Berry B.C., Zheng S., Carroll R.S., Johnson M.D., Yang H.W. (2023). Heterozygous FOXJ1 Mutations Cause Incomplete Ependymal Cell Differentiation and Communicating Hydrocephalus. Cell. Mol. Neurobiol..

[bib28] Ishikawa T. (2017). Axoneme Structure from Motile Cilia. Cold Spring Harbor Perspect. Biol..

[bib29] Ji W., Tang Z., Chen Y., Wang C., Tan C., Liao J., Tong L., Xiao G. (2022). Ependymal Cilia: Physiology and Role in Hydrocephalus. Front. Mol. Neurosci..

[bib31] Johansson C.B., Momma S., Clarke D.L., Risling M., Lendahl U., Frisén J. (1999). Identification of a NSC in the adult mammalian central nervous system. Cell.

[bib32] Kaplani K., Lalioti M.-E., Vassalou S., Lokka G., Parlapani E., Kritikos G., Lygerou Z., Taraviras S. (2024). Ependymal cell lineage reprogramming as a potential therapeutic intervention for hydrocephalus. EMBO Mol. Med..

[bib33] Karamitros D., Kotantaki P., Lygerou Z., Veiga-Fernandes H., Pachnis V., Kioussis D., Taraviras S. (2010). Differential geminin requirement for proliferation of thymocytes and mature T cells. J. Immunol..

[bib34] Karamitros D., Kotantaki P., Lygerou Z., Kioussis D., Taraviras S. (2011). T cell proliferation and homeostasis: an emerging role for the cell cycle inhibitor geminin. Crit. Rev. Immunol..

[bib35] Karamitros D., Patmanidi A.L., Kotantaki P., Potocnik A.J., Bähr-Ivacevic T., Benes V., Lygerou Z., Kioussis D., Taraviras S. (2015). Geminin deletion increases the number of fetal hematopoietic stem cells by affecting the expression of key transcription factors. Development.

[bib36] Konstantinidou C., Taraviras S., Pachnis V. (2016). Geminin prevents DNA damage in vagal neural crest cells to ensure normal enteric neurogenesis. BMC Biol..

[bib37] Kumar V., Umair Z., Kumar S., Goutam R.S., Park S., Kim J. (2021). The regulatory roles of motile cilia in CSF circulation and hydrocephalus. Fluids Barriers CNS.

[bib38] Kyrousi C., Arbi M., Pilz G.-A., Pefani D.-E., Lalioti M.-E., Ninkovic J., Götz M., Lygerou Z., Taraviras S. (2015). Mcidas and GemC1 are key regulators for the generation of multiciliated ependymal cells in the adult neurogenic niche. Dev. Camb. Engl..

[bib39] Kyrousi C., Lygerou Z., Taraviras S. (2017). How a radial glial cell decides to become a multiciliated ependymal cell. Glia.

[bib40] Lalioti M.-E., Arbi M., Loukas I., Kaplani K., Kalogeropoulou A., Lokka G., Kyrousi C., Mizi A., Georgomanolis T., Josipovic N., Taraviras S. (2019). GemC1 governs multiciliogenesis through direct interaction with and transcriptional regulation of p73. J. Cell Sci..

[bib41] Lalioti M.-E., Kaplani K., Lokka G., Georgomanolis T., Kyrousi C., Dong W., Dunbar A., Parlapani E., Damianidou E., Spassky N. (2019). GemC1 is a critical switch for neural stem cell generation in the postnatal brain. Glia.

[bib42] Lewis M., Stracker T.H. (2021). Transcriptional regulation of multiciliated cell differentiation. Semin. Cell Dev. Biol..

[bib43] Lewis M., Terré B., Knobel P.A., Cheng T., Lu H., Attolini C.S.-O., Smak J., Coyaud E., Garcia-Cao I., Sharma S. (2023). GEMC1 and MCIDAS interactions with SWI/SNF complexes regulate the multiciliated cell-specific transcriptional program. Cell Death Dis..

[bib44] Li Y., Chen J., Chopp M. (2002). Cell proliferation and differentiation from ependymal, subependymal and choroid plexus cells in response to stroke in rats. J. Neurol. Sci..

[bib45] Lim D.A., Alvarez-Buylla A. (2016). The Adult Ventricular–Subventricular Zone (V-SVZ) and Olfactory Bulb (OB) Neurogenesis. Cold Spring Harbor Perspect. Biol..

[bib46] Lim D.A., Alvarez-Buylla A. (2014). Adult NSCs stake their ground. Trends Neurosci..

[bib47] Liu Z., Nguyen Q.P.H., Nanjundappa R., Delgehyr N., Megherbi A., Doherty R., Thompson J., Jackson C., Albulescu A., Heng Y.M. (2020). Super-resolution Microscopy and FIB-SEM Imaging Reveal Parental Centriole-Derived, Hybrid Cilium in Mammalian Multiciliated Cells. Dev. Cell.

[bib48] Luo Y., Coskun V., Liang A., Yu J., Cheng L., Ge W., Shi Z., Zhang K., Li C., Cui Y. (2015). Single-Cell Transcriptome Analyses Reveal Signals to Activate Dormant Neural Stem Cells. Cell.

[bib49] Ma L., Quigley I., Omran H., Kintner C. (2014). Multicilin drives centriole biogenesis via E2f proteins. Genes Dev..

[bib50] Mahjoub M.R., Nanjundappa R., Harvey M.N. (2022). Development of a multiciliated cell. Curr. Opin. Cell Biol..

[bib51] Marcy G., Foucault L., Babina E., Capeliez T., Texeraud E., Zweifel S., Heinrich C., Hernandez-Vargas H., Parras C., Jabaudon D., Raineteau O. (2023). Single-cell analysis of the postnatal dorsal V-SVZ reveals a role for Bmpr1a signaling in silencing pallial germinal activity. Sci. Adv..

[bib52] Meletis K., Barnabé-Heider F., Carlén M., Evergren E., Tomilin N., Shupliakov O., Frisén J. (2008). Spinal Cord Injury Reveals Multilineage Differentiation of Ependymal Cells. PLoS Biol..

[bib53] Merkle F.T., Tramontin A.D., García-Verdugo J.M., Alvarez-Buylla A. (2004). Radial glia give rise to adult neural stem cells in the subventricular zone. Proc. Natl. Acad. Sci..

[bib55] Meunier A., Azimzadeh J. (2016). Multiciliated Cells in Animals. Cold Spring Harbor Perspect. Biol..

[bib56] Milich L.M., Choi J.S., Ryan C., Cerqueira S.R., Benavides S., Yahn S.L., Tsoulfas P., Lee J.K. (2021). Single-cell analysis of the cellular heterogeneity and interactions in the injured mouse spinal cord. J. Exp. Med..

[bib57] Mirzadeh Z., Kusne Y., Duran-Moreno M., Cabrales E., Gil-Perotin S., Ortiz C., Chen B., Garcia-Verdugo J.M., Sanai N., Alvarez-Buylla A. (2017). Bi- and uniciliated ependymal cells define continuous floor-plate-derived tanycytic territories. Nat. Commun..

[bib58] Mirzadeh Z., Merkle F.T., Soriano-Navarro M., García-Verdugo J.M., Alvarez-Buylla A. (2008). NSCs confer unique pinwheel architecture to the ventricular surface in neurogenic regions of the adult brain. Cell Stem Cell.

[bib59] Misson J.P., Edwards M.A., Yamamoto M., Caviness V.S. (1988). Identification of radial glial cells within the developing murine central nervous system: studies based upon a new immunohistochemical marker. Brain Res. Dev. Brain Res..

[bib60] Muthusamy N., Brumm A., Zhang X., Carmichael S.T., Ghashghaei H.T. (2018). Foxj1 expressing ependymal cells do not contribute new cells to sites of injury or stroke in the mouse forebrain. Sci. Rep..

[bib61] Nomura T., Göritz C., Catchpole T., Henkemeyer M., Frisén J. (2010). EphB Signaling Controls Lineage Plasticity of Adult NSC Niche Cells. Cell Stem Cell.

[bib62] Obernier K., Alvarez-Buylla A. (2019). Neural stem cells: origin, heterogeneity and regulation in the adult mammalian brain. Development.

[bib63] Ohata S., Nakatani J., Herranz-Pérez V., Cheng J., Belinson H., Inubushi T., Snider W.D., García-Verdugo J.M., Wynshaw-Boris A., Alvarez-Buylla A. (2014). Loss of Dishevelleds disrupts planar polarity in ependymal motile cilia and results in hydrocephalus. Neuron.

[bib64] Ortiz-Álvarez G., Daclin M., Shihavuddin A., Lansade P., Fortoul A., Faucourt M., Clavreul S., Lalioti M.-E., Taraviras S., Hippenmeyer S. (2019). Adult Neural Stem Cells and Multiciliated Ependymal Cells Share a Common Lineage Regulated by the Geminin Family Members. Neuron.

[bib65] Patmanidi A.L., Champeris Tsaniras S., Karamitros D., Kyrousi C., Lygerou Z., Taraviras S. (2017). Concise Review: Geminin-A Tale of Two Tails: DNA Replication and Transcriptional/Epigenetic Regulation in Stem Cells. Stem Cell..

[bib66] Pefani D.-E., Dimaki M., Spella M., Karantzelis N., Mitsiki E., Kyrousi C., Symeonidou I.-E., Perrakis A., Taraviras S., Lygerou Z. (2011). Idas, a novel phylogenetically conserved geminin-related protein, binds to geminin and is required for cell cycle progression. J. Biol. Chem..

[bib67] Pozniak C.D., Barnabé-Heider F., Rymar V.V., Lee A.F., Sadikot A.F., Miller F.D. (2002). p73 is required for survival and maintenance of CNS neurons. J. Neurosci..

[bib68] Redmond S.A., Figueres-Oñate M., Obernier K., Nascimento M.A., Parraguez J.I., López-Mascaraque L., Fuentealba L.C., Alvarez-Buylla A. (2019). Development of Ependymal and Postnatal Neural Stem Cells and Their Origin from a Common Embryonic Progenitor. Cell Rep..

[bib69] Ren Y., Ao Y., O’Shea T.M., Burda J.E., Bernstein A.M., Brumm A.J., Muthusamy N., Ghashghaei H.T., Carmichael S.T., Cheng L., Sofroniew M.V. (2017). Ependymal cell contribution to scar formation after spinal cord injury is minimal, local and dependent on direct ependymal injury. Sci. Rep..

[bib70] Robson E.A., Dixon L., Causon L., Dawes W., Benenati M., Fassad M., Hirst R.A., Kenia P., Moya E.F., Patel M. (2020). Hydrocephalus and diffuse choroid plexus hyperplasia in primary ciliary dyskinesia-related MCIDAS mutation. Neurol. Genet..

[bib71] Rodrigo Albors A., Singer G.A., Llorens-Bobadilla E., Frisén J., May A.P., Ponting C.P., Storey K.G. (2023). An ependymal cell census identifies heterogeneous and ongoing cell maturation in the adult mouse spinal cord that changes dynamically on injury. Dev. Cell.

[bib72] Rodriguez-Jimenez F.J., Jendelova P., Erceg S. (2023). The activation of dormant ependymal cells following spinal cord injury. Stem Cell Res. Ther..

[bib73] Ryu H., Lee H., Lee J., Noh H., Shin M., Kumar V., Hong S., Kim J., Park S. (2021). The molecular dynamics of subdistal appendages in multi-ciliated cells. Nat. Commun..

[bib75] Shah P.T., Stratton J.A., Stykel M.G., Abbasi S., Sharma S., Mayr K.A., Koblinger K., Whelan P.J., Biernaskie J. (2018). Single-Cell Transcriptomics and Fate Mapping of Ependymal Cells Reveals an Absence of Neural Stem Cell Function. Cell.

[bib76] Shahid U., Singh P. (2018). Emerging Picture of Deuterosome-Dependent Centriole Amplification in MCCs. Cells.

[bib77] Siyahhan B., Knobloch V., de Zélicourt D., Asgari M., Schmid Daners M., Poulikakos D., Kurtcuoglu V. (2014). Flow induced by ependymal cilia dominates near-wall cerebrospinal fluid dynamics in the lateral ventricles. J. R. Soc. Interface.

[bib78] Soares H., Carmona B., Nolasco S., Viseu Melo L., Gonçalves J. (2019). Cilia Distal Domain: Diversity in Evolutionarily Conserved Structures. Cells.

[bib79] Spassky N., Merkle F.T., Flames N., Tramontin A.D., García-Verdugo J.M., Alvarez-Buylla A. (2005). Adult ependymal cells are postmitotic and are derived from radial glial cells during embryogenesis. J. Neurosci..

[bib80] Spassky N., Meunier A. (2017). The development and functions of multiciliated epithelia. Nat. Rev. Mol. Cell Biol..

[bib81] Spella M., Kyrousi C., Kritikou E., Stathopoulou A., Guillemot F., Kioussis D., Pachnis V., Lygerou Z., Taraviras S. (2011). Geminin regulates cortical progenitor proliferation and differentiation. Stem Cell..

[bib82] Stenudd M., Sabelström H., Llorens-Bobadilla E., Zamboni M., Blom H., Brismar H., Zhang S., Basak O., Clevers H., Göritz C. (2022). Identification of a discrete subpopulation of spinal cord ependymal cells with neural stem cell properties. Cell Rep..

[bib83] Stratton J.A., Shah P., Sinha S., Crowther E., Biernaskie J. (2019). A tale of two cousins: ECs, quiescent NSCs and potential mechanisms driving their functional divergence. FEBS J..

[bib84] Stubbs J.L., Oishi I., Izpisúa Belmonte J.C., Kintner C. (2008). The Forkhead protein, FoxJ1, specifies node-like cilia in Xenopus and Zebrafish embryos. Nat. Genet..

[bib85] Terré B., Piergiovanni G., Segura-Bayona S., Gil-Gómez G., Youssef S.A., Attolini C.S.O., Wilsch-Bräuninger M., Jung C., Rojas A.M., Marjanović M. (2016). GEMC1 is a critical regulator of multiciliated cell differentiation. EMBO J..

[bib86] Urbán N., Cheung T.H. (2021). Stem cell quiescence: the challenging path to activation. Dev. Camb. Engl..

[bib87] Yamada S., Ishikawa M., Nozaki K. (2021). Exploring mechanisms of ventricular enlargement in idiopathic normal pressure hydrocephalus: a role of cerebrospinal fluid dynamics and motile cilia. Fluids Barriers CNS.

[bib88] Yu X., Ng C.P., Habacher H., Roy S. (2008). Foxj1 transcription factors are master regulators of the motile ciliogenic program. Nat. Genet..

[bib89] Zhao H., Chen Q., Fang C., Huang Q., Zhou J., Yan X., Zhu X. (2019). Parental centrioles are dispensable for deuterosome formation and function during basal body amplification. EMBO Rep..

